# Comparison of Laparoscopic-Assisted Transversus Abdominis Plane Block and Trocar-Site Local Anesthetic Infiltration After Laparoscopic Appendectomy: A Randomized Controlled Trial

**DOI:** 10.7759/cureus.118156

**Published:** 2026-10-19

**Authors:** İksan Taşdelen, Fatih Kilic, Kemal Memişoğlu, Ahmet Yalin Iscan, Bülent Kaya, Arzu Yildirim Ar

**Affiliations:** 1 General Surgery, Fatih Sultan Mehmet Training and Research Hospital, Istanbul, TUR; 2 Surgery, Fatih Sultan Mehmet Training and Research Hospital, Istanbul, TUR; 3 General Surgery, Istanbul University - Çapa, Istanbul, TUR; 4 General Surgery, Istanbul Rumeli University, Istanbul, TUR; 5 Anesthesiology and Reanimation, Fatih Sultan Mehmet Training and Research Hospital, Istanbul, TUR

**Keywords:** abdominal wall block, laparoscopic appendectomy, local anesthetic infiltration, multimodal analgesia, opioid-sparing analgesia, postoperative pain, transversus abdominis plane block

## Abstract

Background and objective: Abdominal wall blocks are increasingly used for pain control following laparoscopic appendectomy. This study aimed to compare the analgesic efficacy of a transversus abdominis plane (TAP) block performed by the surgical team under direct laparoscopic visualization with trocar-site infiltration using an equal total dose of local anesthetic (LA).

Materials and methods: Sixty patients who underwent laparoscopic appendectomy for uncomplicated acute appendicitis between August 2017 and January 2018 were prospectively randomized in a 1:1 ratio into two groups. In the TAP group (n = 30), 15 mL of 0.25% bupivacaine was administered bilaterally under direct laparoscopic visualization before appendectomy. In the LA group (n = 30), 5 mL of 0.5% bupivacaine was infiltrated at each trocar site before incision. The total bupivacaine dose was standardized to 75 mg in both groups. Pain intensity was measured using the visual analog scale (VAS) at postoperative hours 0, 2, 6, 12, and 24. The two groups were compared in terms of rescue analgesic consumption; time to first mobilization, first passage of flatus, and initiation of oral intake; nausea and vomiting; complications; and length of hospital stay.

Results: The groups were comparable in terms of age, sex, body mass index, and American Society of Anesthesiologists (ASA) score (all p > 0.05). VAS scores were significantly lower in the TAP group at all measured time points (hour 0, p = 0.005; hour 2, p = 0.022; hour 6, p = 0.020; hour 12, p = 0.020; and hour 24, p = 0.001; effect sizes, r = 0.34-0.49). In the TAP group, 19 of 30 patients (63.3%) required no rescue analgesia during the first 24 hours, compared with 11 of 30 (36.7%) in the LA group (p = 0.039); the total number of nonsteroidal anti-inflammatory drug (NSAID) doses per patient was significantly lower in the TAP group (p = 0.023). Although opioid requirements were numerically lower in the TAP group, the difference did not reach statistical significance (2/30, 6.7% vs. 7/30, 23.3%; p = 0.071). Oral intake was initiated earlier in the TAP group (median, 8.0 vs. 10.0 hours; p = 0.018). The median length of hospital stay was 24.0 hours in the TAP group and 32.0 hours in the LA group (p = 0.002), and the proportions of patients discharged within 24 hours were 16 of 30 (53.3%) and 3 of 30 (10.0%), respectively (p < 0.001). No differences were observed between the groups in mobilization, passage of flatus, or the frequency of nausea and vomiting. No complications related to block administration were observed.

Conclusions: A surgeon-performed TAP block under laparoscopic visualization provides lower pain scores and reduces analgesic consumption during the first 24 hours following laparoscopic appendectomy compared with trocar-site infiltration using an equal dose of LA. As it requires neither additional equipment nor ultrasonographic expertise, this technique represents a feasible option in emergency surgical practice.

## Introduction

Acute appendicitis is the most common intra-abdominal condition encountered in emergency surgical practice, with an estimated lifetime risk of surgery of approximately 7% [1]. Laparoscopic appendectomy is recommended as the standard approach in centers with appropriate equipment and expertise in the current World Society of Emergency Surgery (WSES) Jerusalem guidelines [2].

Despite the advantages of the laparoscopic approach, postoperative pain is not eliminated entirely. Pain following laparoscopic appendectomy is multifactorial and includes somatic incisional pain at trocar entry sites, visceral pain originating from the appendectomy site, and diaphragmatic irritation related to pneumoperitoneum. A substantial proportion of patients continue to report pain during the postoperative period. Inadequately controlled pain affects not only patient comfort but also pulmonary and cardiac complication rates, the time to mobilization and initiation of oral intake, and consequently the length of hospital stay [3].

Reducing opioid use is a central goal of contemporary perioperative medicine. Opioids contribute to postoperative ileus through nausea and vomiting, sedation, urinary retention, and suppression of intestinal motility; enhanced recovery after surgery (ERAS) protocols define opioid-sparing multimodal analgesia as a core component. In this context, regional blocks have emerged as a means of reducing systemic analgesic requirements and supporting early mobilization and discharge.

Among abdominal wall blocks, the transversus abdominis plane (TAP) block was described by Rafi in 2001 using a loss-of-resistance technique through the triangle of Petit [4] and entered clinical use through the studies of McDonnell et al. [5]. The block targets somatic pain of the anterior abdominal wall by depositing local anesthetic (LA) in the neurofascial plane between the internal oblique and transversus abdominis muscles, thereby blocking the anterior branches of the T7-L1 thoracolumbar nerves. Its adoption accelerated with ultrasound-guided application; however, dependence on an ultrasound device and the operator’s sonographic skill remains a practical barrier, particularly in emergency settings.

In 2011, Magee et al. described a technique in which the block could be performed by the surgeon under direct laparoscopic visualization by observing tenting of the peritoneum over the needle tip [6]. Laparoscopic-assisted TAP (LTAP) block has attracted interest as a surgically compatible alternative because it does not require ultrasound or additional personnel, causes no loss of time, and reduces the risks of intraperitoneal injection and organ injury by allowing direct visualization of the needle tip from the peritoneal aspect.

Conversely, whether abdominal wall blocks provide a genuine advantage over simple trocar-site LA infiltration remains controversial. Trocar-site infiltration is inexpensive, rapid, easy to learn, and nearly risk-free; if it provides similar analgesia, routine use of a more complex block becomes difficult to justify. In a substantial proportion of studies comparing these techniques, however, the total LA dose administered to the groups was not equalized. Consequently, it remains unclear whether observed differences result from the anatomical target of the block or merely from a higher drug dose.

In this study, we aimed to compare the analgesic efficacy of a preemptive bilateral TAP block performed by the surgical team under direct laparoscopic visualization with trocar-site infiltration using an equal total dose of LA in patients undergoing laparoscopic appendectomy for uncomplicated acute appendicitis.

## Materials and methods

This prospective, randomized, controlled, single-center study was conducted in the Department of General Surgery at the University of Health Sciences, Fatih Sultan Mehmet Training and Research Hospital, between August 16, 2017, and January 5, 2018. Follow-up of the last enrolled patient was completed on January 6, 2018. The study protocol was prepared in accordance with the principles of the Declaration of Helsinki [7] and approved by the Clinical Research Ethics Committee of Fatih Sultan Mehmet Training and Research Hospital (decision no. 2017/26; date: July 27, 2017). Written informed consent was obtained from all patients before surgery. Reporting followed the CONSORT (Consolidated Standards of Reporting Trials) statement. The trial was registered at ClinicalTrials.gov (identifier NCT07849751); registration was retrospective, with the record first submitted to the registry on September 24, 2026, and first posted on September 30, 2026. The registered primary outcome, postoperative pain intensity measured by the visual analog scale (VAS) at 0, 2, 6, 12, and 24 hours, is reported as the primary outcome here, and the secondary outcomes reported in this article are those listed in the registered study record. The study protocol, including these outcome measures, had been approved by the institutional ethics committee on July 27, 2017, before the enrolment of the first patient on August 16, 2017. The trial was not registered prospectively. The protocol was developed and approved as an institutional specialty thesis protocol, and prospective registration was not undertaken at that time. The registry record was created in preparation for the present report; the outcome measures, eligibility criteria, interventions, enrolment period, and sample size entered in the registry were transcribed from the ethics committee-approved protocol and from the original dataset, both of which were retained unchanged.

Patients scheduled for laparoscopic appendectomy with a preliminary diagnosis of acute appendicitis were enrolled. All patients underwent preoperative computed tomography as part of the routine diagnostic workup. Patients whose imaging demonstrated perforation or a periappendiceal phlegmon were classified as having complicated appendicitis and were not enrolled. Patients who were aged 18-65 years, had American Society of Anesthesiologists (ASA) physical status I-II, had imaging consistent with uncomplicated acute appendicitis, were scheduled for laparoscopic appendectomy, and were willing to provide written informed consent were included. Patients with a known allergy to LA agents; a concomitant systemic disease such as diabetes mellitus, chronic kidney failure, or bleeding diathesis; active infection at the block injection site; regular analgesic use for chronic pain; or alcohol or narcotic substance dependence were excluded. Intraoperative identification of perforated or phlegmonous appendicitis and conversion from laparoscopy to open surgery were additional predefined exclusion criteria, retained as safeguards after randomization. As complicated appendicitis had already been excluded on preoperative imaging, neither criterion was met in any patient, and no participant was excluded after group allocation.

The allocation sequence consisted of a fixed set of 60 opaque, sealed envelopes prepared in advance by the investigators and shuffled before the start of the study, with 30 assigned to the TAP group and 30 to the LA group. The envelopes were not sequentially numbered. The deck was kept by the operating surgeon. After eligibility had been confirmed and written informed consent obtained, the patient drew one envelope from the shuffled deck; the surgeon then opened the envelope and recorded the assignment. Patients who drew a white envelope received a TAP block under laparoscopic visualization (TAP group), and those who drew a black envelope received bupivacaine infiltration at the trocar entry sites (LA group). Because the draw took place only after eligibility and consent had been established, the surgeon could neither influence which patient received which intervention nor alter eligibility on the basis of the allocation. Allocation concealment was nevertheless partial: the sequence was neither generated nor held by a party independent of the study team, the envelopes were not sequentially numbered, and because allocation was by drawing without replacement from a fixed deck, the composition of the remaining envelopes, and therefore the probability of each subsequent assignment, became progressively more predictable as recruitment proceeded. This procedure does not meet the standard of sequentially numbered, opaque, sealed envelopes or of centralized allocation, and this is acknowledged as a limitation. The operating surgeon could not be blinded because of the nature of the intervention. Patients were not informed of the correspondence between the color of the envelope they drew and the technique to be applied. During the postoperative period, the ward team that obtained VAS measurements and decided on rescue analgesia was unaware of group allocation. Statistical analysis was performed with group identity masked. Thus, the study was conducted as a single-blind (assessor-blinded) trial.

TAP group: TAP block under laparoscopic visualization

After induction of anesthesia and intubation, a 1-cm midline infraumbilical incision was made, and the abdomen was entered using the direct trocar technique. After establishing pneumoperitoneum, the operating table was placed in the reverse Trendelenburg position and tilted to the contralateral side. A 22-G spinal needle was advanced perpendicular to the skin through the triangle of Petit, bounded by the subcostal line, iliac crest, and lateral border of the latissimus dorsi muscle (Figure 1, Panel a). The needle tip was directly observed over the peritoneum using the laparoscopic camera to confirm the correct depth; when peritoneal tenting was observed (Figure 1, Panel b), the needle was withdrawn approximately 0.5-1 cm and considered positioned in the neurofascial plane between the internal oblique and transversus abdominis muscles. After negative aspiration, 15 mL of 0.25% bupivacaine was injected slowly; the formation of a smooth, homogeneous bulge in the peritoneum during injection was considered evidence of block success. The same procedure was repeated on the contralateral side after tilting the table in the opposite direction. The total bupivacaine dose was 30 mL of 0.25% solution (75 mg).

**Figure 1:**
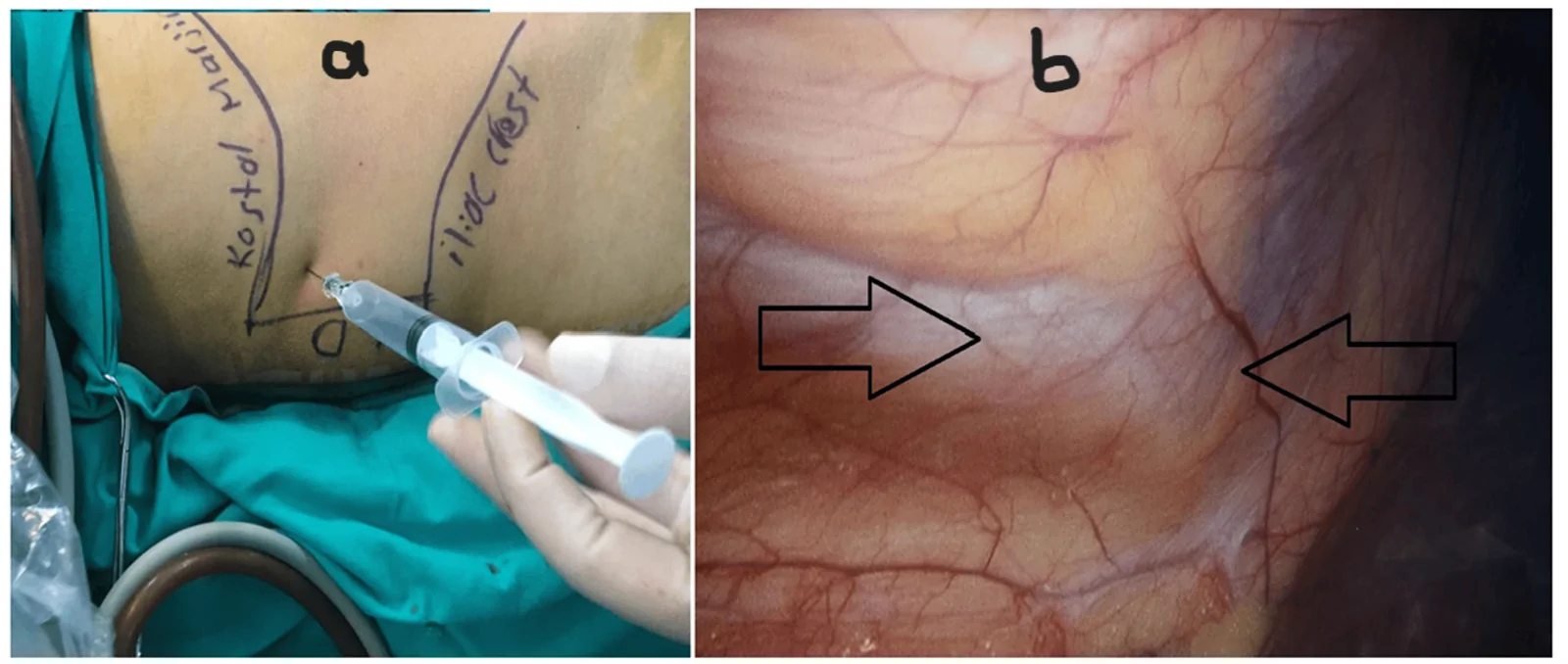
(a) Triangle of Petit and (b) laparoscopic view of peritoneal tenting during injection

LA group: trocar-site LA infiltration

Before skin incision, 5 mL of 0.5% bupivacaine was infiltrated into each of the three trocar entry sites, including the camera trocar site, encompassing the skin, subcutaneous tissue, and fascial layers. The total bupivacaine dose was 15 mL of 0.5% solution (75 mg). Thus, the total LA dose was standardized to 75 mg in both groups, and the toxic dose threshold was not approached in any patient.

Standard three-port laparoscopic appendectomy was performed in both groups. A 10-mm infraumbilical camera trocar, a 10-mm working trocar in the left lower quadrant through the bikini line, and a 5-mm working trocar in the right lower quadrant were inserted. The patient was then placed in the Trendelenburg position, and the table was tilted 15 degrees to the left. Trocar placement sites are shown in Figure 2. The appendix was placed under traction, and the mesoappendix was divided using an energy device; two 2-0 polypropylene endoloops were placed at the base of the appendix, and the appendectomy was completed. In all cases, the appendix was removed from the abdomen using an endobag. Drain use was left to the surgeon’s intraoperative judgment. Wound closure was standardized and identical in all patients: the fascia was closed with 0 absorbable suture at the two 10-mm trocar sites, the 5-mm site was not closed at the fascial level, and the skin was closed at all sites with 3-0 non-absorbable polypropylene vertical mattress sutures.

**Figure 2:**
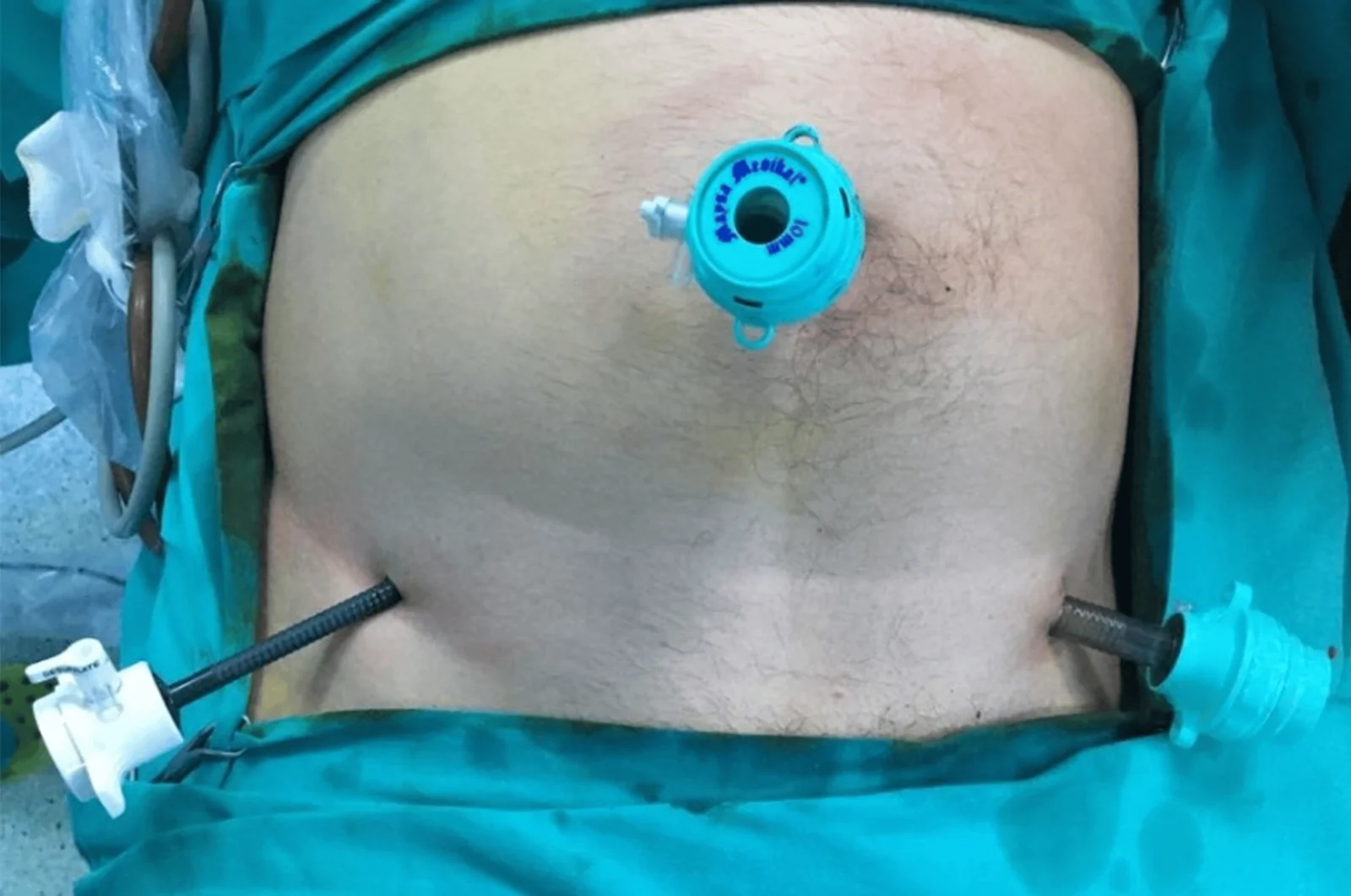
Trocar placement sites

Before completion of surgery, all patients in both groups received intravenous paracetamol 1,000 mg and tramadol 1 mg/kg as components of standardized multimodal analgesia.

The primary outcome was pain intensity at rest, measured using the VAS at postoperative hours 0, 2, 6, 12, and 24. The protocol did not designate a single time point as the primary endpoint; the five measurements were prespecified as a set, and the primary comparison was accordingly made both at each individual time point and across the whole observation period using a mixed analysis of variance. The use of the scale was explained to all patients preoperatively, and a printed 100-mm visual scale was shown to each patient before surgery and at each in-hospital assessment.

Rescue analgesic consumption (type, dose, and timing); time to first mobilization, first passage of flatus, and initiation of oral intake; postoperative nausea and vomiting; operative duration; complications; and length of hospital stay were recorded.

Rescue analgesia was administered using a stepwise protocol. Patients with VAS > 3 first received an intravenous infusion of tenoxicam 20 mg. Patients whose follow-up VAS remained >3 despite nonsteroidal anti-inflammatory drug (NSAID) administration or whose VAS again exceeded 3 within 12 hours received a second-line infusion of tramadol 100 mg. Nineteen patients (16 in the TAP group and three in the LA group) were discharged within 24 hours of surgery, and their 24-hour assessment was therefore conducted by telephone. These calls were made by the ward physician responsible for postoperative follow-up, who was unaware of group allocation. As a visual scale cannot be displayed over the telephone, patients were reminded of the 0-10 scale that had been explained to them preoperatively and used at each in-hospital assessment, and were asked to report their current pain as a number on that scale. Therefore, the measurement took the form of a verbal numerical rating rather than a visual analog rating in these patients. No patient was lost to follow-up.

Statistical analysis

Analyses were performed using SPSS version 20.0 (IBM Corp., Armonk, NY). The normality of variable distributions was assessed using visual methods (histograms and probability plots) and analytical methods (Kolmogorov-Smirnov and Shapiro-Wilk tests). Because VAS scores, numbers of analgesic doses, and time variables were not normally distributed, descriptive statistics are presented as both mean ± standard deviation and median (interquartile range (IQR)), and comparisons were performed using the Mann-Whitney U test. Categorical variables were compared using Pearson’s chi-square test or Fisher’s exact test. Repeated VAS measurements over time were evaluated using repeated-measures analysis of variance with Greenhouse-Geisser correction. Associations between continuous variables were examined using Spearman correlation analysis. Test statistics are reported alongside p-values throughout: the Mann-Whitney U statistic with the corresponding standardized z value is reported for rank-based comparisons, and the Pearson chi-square statistic with its degrees of freedom is reported for categorical comparisons. Effect sizes are reported as the rank-biserial correlation coefficient (r) for Mann-Whitney comparisons, Cramér’s V for chi-square comparisons, and partial eta squared (ηp²) for analysis of variance. In addition to the time-point-by-time-point comparisons, a two-way mixed analysis of variance (within-subject factor: time; between-subject factor: group) was performed on the VAS scores, with Greenhouse-Geisser correction applied because the assumption of sphericity was violated (Mauchly’s W = 0.691, p = 0.012; ε = 0.852). A p-value < 0.05 was considered statistically significant.

The sample size was determined by an a priori power analysis based on the primary outcome. Assuming a clinically meaningful between-group difference of 1.1 points in VAS score (δ) with a standard deviation of 1.5 points (σ), the required number of patients per group (n) was calculated using the standard formula for the comparison of two independent means (Figure 3).

**Figure 3:**
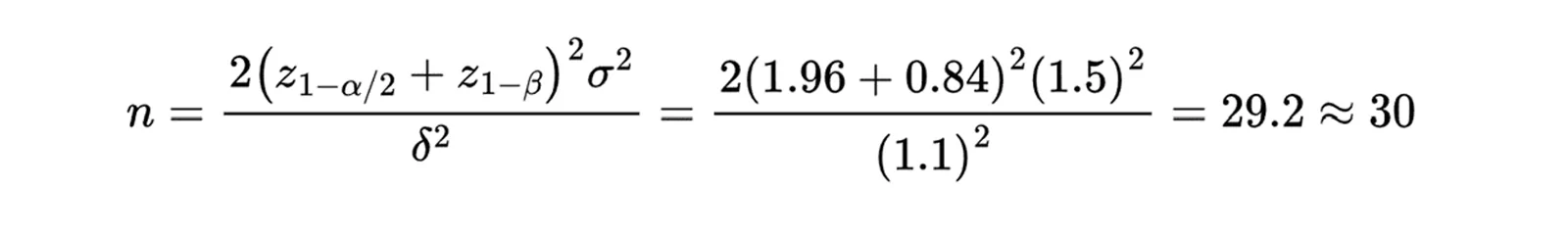
Sample size calculation

With α = 0.05 (two-sided) and 80% power (β = 0.20), a minimum of 30 patients per group was required; accordingly, 60 patients were enrolled. The calculation was performed for a single two-group comparison of VAS scores and did not incorporate the repeated nature of the five measurements; no inflation factor for repeated measures and no adjustment for multiple comparisons across time points were applied. The study was therefore powered to detect a difference of the stated magnitude at an individual time point, and the time-point analyses should be read as a set of prespecified comparisons rather than as independently confirmatory tests.

To address the imbalance in operative duration between the groups, three prespecified sensitivity analyses were performed: Spearman correlations between operative duration and each outcome, overall and within each group; a rank-based analysis of covariance of the VAS scores with group and operative duration as covariates; and a comparison restricted to the range of operative duration common to both groups. In preparation for this report, all analyses were repeated on the original dataset, and the values presented here reflect that reanalysis.

## Results

Sixty patients meeting the study criteria underwent laparoscopic appendectomy during the study period. The requirement for rescue analgesia was lower in the TAP group. Nineteen of 30 patients (63.3%) in the TAP group required no rescue analgesia during the first 24 hours, compared with 11 of 30 (36.7%) in the LA group (χ² = 4.27, df = 1, p = 0.039, V = 0.27). The total number of NSAID doses per patient was significantly lower in the TAP group (U = 311.5, z = −2.274, p = 0.023, r = 0.31). In contrast, the distribution of NSAID dose categories did not differ significantly between groups (χ² = 5.60, df = 3, p = 0.133, V = 0.31). Opioids were required by two patients (6.7%) in the TAP group and seven patients (23.3%) in the LA group. Despite this marked numerical difference, neither the difference in the proportion of patients requiring opioids (χ² = 3.27, df = 1, p = 0.071, V = 0.23) nor that in the total number of opioid doses per patient (U = 374.0, z = −1.814, p = 0.070, r = 0.17) reached statistical significance (Table 1).

**Table 1:** Comparison of rescue analgesic consumption between groups *Mann-Whitney U test, **Pearson chi-square test. NSAID: Nonsteroidal anti-inflammatory drug; IQR: Interquartile range; U: Mann-Whitney U statistic; z: Standardized test statistic; χ²: Pearson chi-square statistic; df: Degrees of freedom; r: Rank-biserial correlation; V: Cramér’s V.

Variable	TAP group (n = 30)	LA group (n = 30)	Test statistic	p	Effect size
No rescue analgesia required, n (%)	19 (63.3)	11 (36.7)	χ² = 4.27, df = 1	0.039**	V = 0.27
Number of NSAID doses, n (%)			χ² = 5.60, df = 3	0.133**	V = 0.31
0 doses	19 (63.3)	11 (36.7)			
1 dose	10 (33.3)	14 (46.7)			
2 doses	1 (3.3)	4 (13.3)			
3 doses	0 (0.0)	1 (3.3)			
Total NSAID doses per patient, median (IQR)	0 (0-1)	1 (0-1)	U = 311.5, z = −2.274	0.023*	r = 0.31
Patients requiring opioids, n (%)	2 (6.7)	7 (23.3)	χ² = 3.27, df = 1	0.071**	V = 0.23
Total opioid doses per patient, median (IQR)	0 (0-0)	0 (0-0)	U = 374.0, z = −1.814	0.070*	r = 0.17

Spearman correlation analysis showed a significant moderate positive association between total NSAID and total opioid consumption (ρ = 0.415, n = 60, p = 0.001), indicating that opioid requirements increased as the need for first-line analgesia increased. Length of hospital stay was not significantly associated with analgesic consumption (NSAIDs, ρ = 0.246, p = 0.058; opioids, ρ = 0.136, p = 0.299).

Oral intake was initiated significantly earlier in the TAP group (median, 8.0 vs. 10.0 hours; p = 0.018). In contrast, no significant between-group differences were observed in time to first mobilization (p = 0.227) or first passage of flatus (p = 0.235); in both groups, patients were mobilized at an average of six hours postoperatively. The distribution of length of hospital stay differed significantly between groups (median, 24.0 vs. 32.0 hours; U = 242.5, z = −3.087, p = 0.002, r = 0.46). The means, however, were similar (32.4 vs. 33.0 hours) because two outliers in the TAP group with stays of 102 and 144 hours, both of whom developed complications, increased the mean. The more clinically interpretable measure, discharge within 24 hours, occurred in 16 of 30 patients (53.3%) in the TAP group and three of 30 patients (10.0%) in the LA group (χ² = 13.02, df = 1, p < 0.001, V = 0.47) (Table 2).

**Table 2:** Comparison of postoperative outcomes between groups *Mann-Whitney U test, **Pearson chi-square test (Fisher’s exact test p = 0.001 for discharge within 24 hours). IQR: Interquartile range; SD: Standard deviation; U: Mann-Whitney U statistic; z: Standardized test statistic; χ²: Pearson chi-square statistic; df: Degrees of freedom; r: Rank-biserial correlation; V: Cramér’s V.

Variable	TAP group (n = 30)	LA group (n = 30)	Test statistic	p	Effect size
First mobilization (hours), median (IQR)	6.0 (6.0-6.0)	6.0 (6.0-7.5)	U = 393.0, z = −1.209	0.227*	r = 0.13
First passage of flatus (hours), median (IQR)	10.0 (8.0-12.0)	10.0 (8.0-14.0)	U = 370.5, z = −1.188	0.235*	r = 0.18
Initiation of oral intake (hours), median (IQR)	8.0 (8.0-11.5)	10.0 (8.0-12.0)	U = 295.0, z = −2.360	0.018*	r = 0.34
Length of hospital stay (hours), median (IQR)	24.0 (22.5-29.5)	32.0 (28.0-36.0)	U = 242.5, z = −3.087	0.002*	r = 0.46
Length of hospital stay (hours), mean ± SD	32.43 ± 26.54	33.00 ± 10.54			
Discharge within 24 hours, n (%)	16 (53.3)	3 (10.0)	χ² = 13.02, df = 1	<0.001**	V = 0.47
Nausea/vomiting, n (%)	1 (3.3)	2 (6.7)	χ² = 0.35, df = 1	0.554**	V = 0.08
Drain use, n (%)	6 (20.0)	7 (23.3)	χ² = 0.10, df = 1	0.754**	V = 0.04
Complications, n (%)	4 (13.3)	1 (3.3)	χ² = 1.96, df = 1	0.161**	V = 0.18

Complications in the TAP group included postoperative ileus in two patients, fever in one patient, and acute urinary retention in one patient; postoperative ileus developed in one patient in the LA group. Complication rates did not differ significantly between groups. No block-related complications (hematoma, splenic or hepatic puncture, bowel perforation, or LA systemic toxicity) were observed. No wound infections or complications requiring reoperation were reported.

When patients with drains (n = 13) were compared with those without drains (n = 47), VAS scores were similar during the first 12 hours (all p > 0.05), whereas VAS scores at hour 24 were significantly higher in patients with drains (median, 2.0 vs. 1.0; U = 169.0, z = −2.563, p = 0.010, r = 0.45). Scores at hour 6 were also higher in patients with drains, although the difference did not reach significance (median, 2.0 vs. 1.0; U = 204.5, z = −1.870, p = 0.061, r = 0.33). In the mixed analysis of variance, the drain × time interaction did not reach statistical significance after Greenhouse-Geisser correction (F(3.41, 197.6) = 2.49, p = 0.054, ηp² = 0.04), although pain scores decreased at hour 24 in patients without drains while tending to increase in those with drains. The main effect of drain status was not significant (F(1, 58) = 2.16, p = 0.147, ηp² = 0.04). When patients with drains were assessed separately within each group, VAS scores were higher among patients with drains in the LA group than among those in the TAP group; however, this difference did not reach significance because of the small subgroup sizes (p > 0.05).

## Discussion

In this randomized controlled trial, we demonstrated that a preemptive bilateral TAP block performed by the surgical team under direct laparoscopic visualization provided lower pain scores at all time points and reduced rescue analgesic consumption during the first 24 hours after laparoscopic appendectomy compared with trocar-site infiltration using an equal total dose of LA. Nineteen of 30 patients (63.3%) required no rescue analgesia after the TAP block.

In a 2026 systematic review and meta-analysis, Ammeter et al. identified only four randomized controlled trials, comprising 233 patients, that compared TAP block with trocar-site LA injection in adults undergoing laparoscopic appendectomy [8]. In that analysis, TAP block reduced opioid consumption by 4.50 mg morphine equivalents; pain scores were lower at hours 4, 6, and 12, but no significant differences were found at hours 1 and 2 or over the entire 24-hour period. The certainty of evidence was rated low to very low, and the authors explicitly called for additional randomized trials. Our series of 60 patients makes a quantitatively meaningful contribution to this limited evidence base.

In a multicenter randomized trial conducted at three university hospitals and published in 2026, Daly et al. directly compared LTAP block with port-site infiltration in 167 appendectomy patients [9]. Time-weighted VAS was 1.7 points lower in the TAP group (95% CI, −2.06 to −1.34; p < 0.001); the greatest differences were observed at hours 6 and 12; oxycodone requirements decreased from 1.2 to 0.8 doses (p = 0.034); and the quality-of-life index at one week was higher in the TAP group. Our findings are highly consistent with that study in both direction and magnitude: our mean VAS difference ranged from 0.70 to 1.17 points, and opioid use in the TAP group was reduced to one-third of that in the LA group. Similar findings from two independent centers support the conclusion that the effect is not due to chance.

In a broader context, the meta-analysis by Yu et al. comparing TAP block with LA wound infiltration in lower abdominal surgery reported lower pain scores both at rest and with movement at hour 24 in the TAP group [10]. In a meta-analysis of 51 studies, Brogi et al. showed that TAP block significantly reduced VAS scores at hours 6, 12, and 24 and reduced 24-hour analgesic consumption across various surgical procedures, including appendectomy [11]. The effect is not limited to laparoscopic surgery: Niraj et al. reported that ultrasound-guided TAP (UTAP) block reduced postoperative VAS scores and analgesic consumption in open appendectomy [12], and Peng et al. reported an effect in the same direction in laparoscopic cholecystectomy [13]. Our findings are consistent with this body of evidence.

One of the principal methodological strengths of our study is that the same total bupivacaine dose (75 mg) was administered to both groups. A concentration of 0.25% at 2 × 15 mL was used in the TAP group, whereas 0.5% at 3 × 5 mL was used in the LA group. Thus, volume and concentration differed, but the total dose in milligrams was equal. This design prevents the observed analgesic advantage from being attributed solely to a higher drug dose and strengthens the interpretation that the difference is related to where the LA was deposited, namely, targeting the thoracolumbar nerves within the neurofascial plane. TAP block efficacy is known to depend more on the injected volume than on concentration, and volumes of 15-20 mL per side provide adequate spread in lower abdominal surgery in adults [14]. Our dose selection was consistent with this literature, and no signs of toxicity were observed in any patient.

The preemptive administration of the blocks, before incision and surgical trauma, was also deliberate. In a meta-analysis by Abdallah et al., preincisional TAP block was effective in eight of nine studies examined, whereas postincisional administration was effective in only four of nine studies [15]. Performing the block before central sensitization has developed may explain the observed early advantage.

Application of TAP block under laparoscopic visualization was described by Magee et al., who emphasized that direct visualization of the needle tip from the peritoneal aspect carries a lower risk of iatrogenic complications than the loss-of-resistance technique [6]. Complications such as intrahepatic injection, bowel hematoma, and transient femoral nerve palsy have been reported with both landmark-based and UTAP blocks. No block-related complications occurred in our series, an observation supporting the safety profile of injection under direct visualization. In a study of laparoscopic cholecystectomy by Tihan et al., the method was also reported to be safe, easy to perform, and not to prolong operative duration [16].

Nevertheless, it would be inaccurate to claim that LTAP provides superior analgesia to UTAP block. In a meta-analysis of seven randomized trials comprising 603 patients, Ghani et al. found no significant differences between LTAP and UTAP in pain scores at hours 12 and 24, morphine consumption, time to first analgesic requirement, nausea and vomiting, or return of bowel function after laparoscopic cholecystectomy; only a small difference was observed at hour 6 [17]. As the authors emphasized, the primary advantage of LTAP is logistical rather than analgesic: it requires no additional device, personnel, or procedure time. This advantage is particularly important for appendectomy, which is often performed at night and under emergency conditions in settings where an ultrasound device and an experienced operator may not always be available. The surgeon can complete the block within a few minutes as part of the ongoing laparoscopic procedure, facilitating its integration into emergency surgical practice.

In our study, the median length of hospital stay was eight hours shorter in the TAP group (p = 0.002), and the proportion of patients discharged within 24 hours was more than fivefold higher (16/30, 53.3% vs. 3/30, 10.0%; p < 0.001). Similarly, Keller et al. reported a shorter length of stay in a series of 200 patients undergoing laparoscopic colorectal surgery [18], and Favuzza and Delaney reported the same in a series of 100 patients receiving TAP block as part of an ERAS protocol [19].

This finding should be presented cautiously for two reasons. First, the mean lengths of stay were nearly identical between groups (32.4 vs. 33.0 hours); the difference was evident only in the center of the distribution. This was because two patients with complications in the TAP group, with stays of 102 and 144 hours, increased the mean. Therefore, the result should be interpreted not as “TAP block shortens mean length of stay” but as “TAP block facilitates early discharge in patients with an uncomplicated course.” Second, the meta-analysis by Ammeter et al. found no significant difference in length of hospital stay specifically after appendectomy [8]; the decision to discharge after appendectomy depends not only on pain but also on fever, oral tolerance, laboratory findings, patients’ social circumstances, and clinical practice patterns.

The absence of a between-group difference in the frequency of nausea and vomiting is consistent with studies by the Carney and McDonnell groups [5,20] and with the findings of the meta-analysis by Ammeter et al. [8]. This may be explained by the already low overall opioid exposure in the study population.

An unplanned but noteworthy finding of our study was that drain placement altered the course of pain. VAS scores at hour 24 were significantly higher in patients with drains (p = 0.010), and the pain trajectory tended to diverge from that of patients without drains, although the drain × time interaction did not reach significance after Greenhouse-Geisser correction (p = 0.054): pain decreased at hour 24 in patients without drains but increased in those with drains. This raises the possibility that some late pain may result from mechanical irritation caused by drain traction rather than incisional or visceral sources. Because the subgroups were small, this observation is hypothesis-generating; however, given that drain use in uncomplicated appendectomy is already being questioned, adding pain to this discussion may be meaningful.

The field of regional analgesia has evolved rapidly since the study data were collected. More posterior techniques that may also affect the visceral component, including the erector spinae plane (ESP) block, quadratus lumborum block, and modified thoracoabdominal blocks, have become increasingly common. In a meta-analysis of eight randomized trials comprising 542 patients, Zewdu et al. reported that, compared with TAP block, ESP block for laparoscopic cholecystectomy provided lower resting pain scores at all time points, reduced morphine-equivalent consumption by 3.51 mg, and prolonged the time to first analgesic requirement by approximately 67 minutes [21]. Although these findings show that TAP block is not the only option, ESP block requires ultrasound and cannot be performed by the surgeon from the sterile field. Thus, particularly for emergency, high-volume procedures performed at unpredictable hours, such as appendectomy, TAP block under laparoscopic visualization should be positioned not as the “most powerful block” but as an option offering a pragmatic balance between accessibility and efficacy.

Asymmetries between the two interventions

Two differences between the study arms, beyond the anatomical target of the injection, deserve explicit consideration because each could in principle have influenced the estimated treatment effect.

The first is the timing of administration. Trocar-site infiltration was performed before the skin incision, whereas the TAP block was performed after insertion of the first trocar and establishment of pneumoperitoneum, before appendectomy. The control intervention was thus delivered in a fully preincisional manner, while the experimental intervention was delivered after the first incision had been made. Since preincisional administration is the condition under which abdominal wall blocks have been shown to be most effective [15], this asymmetry would be expected to favor the LA group and therefore to attenuate rather than exaggerate the observed advantage of the TAP block.

The second is the difference in operative duration, which was significantly longer in the LA group (median, 50 vs. 40 minutes). Longer surgery could plausibly increase postoperative pain and thereby inflate the apparent benefit of the block. We examined this in three ways. Operative duration was not associated with pain at any time point except hour 24, where the correlation was weak (ρ = 0.269, p = 0.038). However, within each group, there was no association between operative duration and the 24-hour VAS score (TAP, ρ = −0.061, p = 0.751; LA, ρ = 0.240, p = 0.202). In a rank-based analysis of covariance adjusting for operative duration, the group effect remained significant at hour 0 (p = 0.011) and hour 24 (p = 0.003), while operative duration itself was not an independent predictor (p = 0.634 and p = 0.283, respectively). Finally, in a sensitivity analysis restricted to the overlapping range of operative duration of 35-55 minutes (TAP, n = 20; LA, n = 16), the difference persisted (24-hour VAS: median 1.0 vs. 2.0, p = 0.004). These analyses do not eliminate the imbalance, but they indicate that it is unlikely to account for the difference in pain scores between the groups.

Limitations

First, our study has important limitations. The most significant concerns relate to transparency rather than conduct: the trial was registered retrospectively, in September 2026, almost nine years after the last patient was enrolled. Prospective registration allows the prespecified outcomes of a trial to be publicly verified before any data are examined, and retrospective registration cannot provide that guarantee; however, faithfully, the registry entry reflects the original protocol. Readers therefore cannot confirm from the registry alone that the outcomes reported here were those originally planned or that no additional outcomes were measured but left unreported. What can be documented is that the protocol, including the primary and secondary outcome measures reported in this article, was approved by the institutional ethics committee on July 27, 2017, three weeks before the first patient was enrolled, and that the approved protocol and the original dataset were retained unchanged. We regard this as mitigating but not equivalent to prospective registration, and the findings should be interpreted with this caveat.

Second, allocation concealment was incomplete. The envelopes were opaque and sealed, and the draw took place only after eligibility and consent had been established, which prevented the surgeon from selecting which patient received which intervention. However, the sequence was neither generated nor held by a party independent of the study team, the envelopes were not sequentially numbered, and drawing without replacement from a fixed deck made later allocations progressively more predictable. This procedure does not meet the standard of sequentially numbered, opaque, sealed envelopes or centralized allocation. Block randomization with centralized allocation is recommended for future studies.

Third, because of the small sample size, the clinically noteworthy difference in the proportion of patients requiring opioids, 7 of 30 (23.3%) versus 2 of 30 (6.7%), did not reach statistical significance. Approximately 70 patients per group would be required to detect a difference of this magnitude with 80% power. This result should therefore be interpreted as a difference that could not be demonstrated rather than as the absence of a difference (type II error). Relatedly, the sample size was calculated for a single two-group comparison and did not account for the five repeated VAS measurements, and no adjustment was made for multiple comparisons across time points; the individual time-point results should accordingly be interpreted together with the mixed analysis of variance rather than as independent confirmatory tests.

Fourth, the operating surgeon could not be blinded because of the nature of the intervention, so performance bias cannot be excluded. Relatedly, operative duration was significantly longer in the LA group (median, 50 vs. 40 minutes). This imbalance and the difference in the timing of the two interventions are considered in detail in the preceding section. In brief, neither appears to account for the observed effect, but both remain potential sources of bias that a larger trial with stratified randomization should address.

Fifth, this was a single-center study, which limits the generalizability of the findings to other surgical teams and analgesic protocols.

Sixth, pain was evaluated only at rest using a unidimensional scale. Pain on movement, shoulder pain arising from diaphragmatic irritation, and visceral pain were not measured separately, although the effect of a TAP block is expected to be confined to the somatic component of the anterior abdominal wall. Validated quality-of-recovery instruments such as the QoR-15 and patient satisfaction were not assessed. In addition, 19 patients were discharged within 24 hours, and their final assessment was obtained by telephone, where a visual scale cannot be displayed. For these patients, the measurement was, in effect, a verbal numerical rating rather than a visual analog rating, and this difference in mode of administration may have introduced measurement error at the 24-hour time point.

Seventh, follow-up was limited to 24 hours; postdischarge pain, time to return to work, and the development of chronic postsurgical pain were not evaluated.

Eighth, the study was conducted without a standardized enhanced recovery protocol, and discharge criteria were not predefined. Because the decision to discharge therefore reflected individual clinical judgment, the length-of-stay finding should be regarded as exploratory. No cost-effectiveness analysis was performed.

Finally, the data were collected between 2017 and 2018. The interval between completion of the study and publication was prolonged by the COVID-19 pandemic and by subsequent clinical workload and personal circumstances of the investigators. The dataset, the original statistical output, and the ethics committee-approved protocol were retained unchanged throughout this period, and all analyses were re-executed on the original dataset in preparation for this report. The surgical technique, block technique, and LA agent used remain current, and, as noted above, the body of randomized evidence addressing this specific comparison remains limited [8].

## Conclusions

In this single-center randomized trial of 60 patients undergoing laparoscopic appendectomy, a preemptive bilateral TAP block performed by the surgical team under direct laparoscopic visualization was associated with lower observed pain scores at every measured time point during the first 24 hours and lower rescue NSAID consumption than trocar-site infiltration using an equal total dose of LA. Nineteen of 30 patients (63.3%) in the block group required no rescue analgesia. The block was completed during the existing laparoscopic procedure, without an ultrasound device or an additional operator, and no block-related complications occurred.

These findings should be interpreted as those of a small, single-center trial. The lower requirement for opioids in the block group and the higher proportion of patients discharged within 24 hours were not established with sufficient precision: the opioid difference did not reach statistical significance, and the length-of-stay finding was exploratory because discharge criteria were not predefined. Opioid-sparing efficacy and any effect on early discharge therefore require confirmation. Larger multicenter randomized trials with centralized allocation, standardized recovery and discharge criteria, and assessments of pain on movement and quality of recovery are needed before the LTAP block can be recommended as a routine component of analgesia after appendectomy.
